# Postoperative symptom network analysis in non-small cell lung cancer patients: a cross-sectional study

**DOI:** 10.1186/s12890-025-03711-z

**Published:** 2025-05-20

**Authors:** Sha Zhang, Yao Deng, Xiaorun Xiang, Qianfeng Xu, Lixin Hu, Mei Xia, Lei Liu

**Affiliations:** 1 Department of Thoracic Surgery, The First Affiliated Hospital of the Army Medical University, Chongqing, China; 2 Department of Rheumatology and Immunology, The First Affiliated Hospital of the Army Medical University, Chongqing, China; 3 Department of Cardiac Surgery, The First Affiliated Hospital of the Army Medical University, Chongqing, China; 4 Department of Nursing, The First Affiliated Hospital of the Army Medical University, Chongqing, China

**Keywords:** Non-small cell lung cancer, Symptom network, Shortness of breath, Pain, Network analysis

## Abstract

**Objective:**

To investigate the incidence and severity of symptoms in postoperative non-small-cell lung cancer patients undergoing thoracoscopic surgery, construct a symptom network, and analyze centrality indicators of the network to identify core symptoms and provide a basis for precise symptom management.

**Methods:**

A convenience sampling method was used to select postoperative NSCLC patients from the Department of Thoracic Surgery at the First Affiliated Hospital of Army Medical University between September 2024 and December 2024. The Chinese version of the Anderson Symptom Inventory Core Symptom Module and the revised Lung Cancer-Specific Symptom Module were used to survey the incidence and severity of symptoms. A symptom network was constructed with R software with the EBICgloss function and Spearman correlation analysis, and the centrality indicators were then calculated.

**Results:**

In total, 404 questionnaires were distributed, and 367 valid questionnaires were returned (effective response rate, 90.8%). The top three symptoms in terms of incidence and severity during the postoperative hospitalization of NSCLC patients were pain (100%), fatigue (99%), and shortness of breath (98%). The results of the centrality indicators of the symptom network revealed that the top three symptoms in terms of strength centrality were shortness of breath (rs = 5.44), fatigue (rs = 5.43), and pain (rs = 5.34).

**Conclusion:**

Postoperative NSCLC patients experience various symptoms, with shortness of breath being the core symptom. Targeted intervention strategies are needed to improve the efficiency and accuracy of symptom management, reduce the symptom burden on patients, and increase their quality of life.

**Clinical trial registration:**

Chinese Clinical Trial Registry (NO. ChiCTR2500096720), registered on 5 February 2025, retrospectively registered.

## Introduction

Lung cancer is one of the most common malignant tumors worldwide, with high incidence and mortality. According to data from the International Agency for Research on Cancer (IARC), there were approximately 2.5 million new cases of lung cancer globally in 2022, accounting for 12.4% of all cancer cases, and approximately 1.8 million deaths, accounting for 18.7% of cancer-related deaths, making lung cancer one of the leading causes of cancer-related mortality [1]. In China, according to the 2024 National Cancer Report published by the National Cancer Center (NCC), lung cancer remains the most prevalent and deadly cancer type in the country, accounting for 22% of all malignant tumors in terms of incidence and 28.5% of mortalities in 2022 [2]. These data highlight the significant public health burden of lung cancer not only globally but also within China.

Non-small cell lung cancer (NSCLC) is the most prevalent subtype of lung cancer, accounting for approximately 85% of all lung cancer cases. Among these [3], adenocarcinoma and squamous cell carcinoma are the most common subtypes, accounting for 50% and 20%-30% of NSCLC cases, respectively [4, 5]. For early-stage NSCLC patients, surgery is considered the optimal treatment choice, as it significantly improves patient survival rates [6, 7]. However, even after surgical treatment, patients still experience various symptoms during postoperative recovery. Owing to surgical trauma and the subsequent adverse effects of adjuvant treatments (such as chemotherapy and radiotherapy), patients often experience significant physical and psychological symptoms, which severely affect the patient’s mood and postoperative quality of life [8]. In particular, several studies have shown that postoperative NSCLC patients typically experience up to 11 distressing symptoms, including cough, pain, shortness of breath, and fatigue, all of which significantly affect mood and quality of life [9]. Furthermore, more than half of lung cancer patients still experience 1 to 5 core symptoms at the time of discharge [10], and patients receiving immunotherapy are more likely to experience up to 47 different symptoms [11]. The coexistence of these symptoms further exacerbates the overall symptom burden on patients.

Symptom management is the cornerstone of healthcare and an important part of cancer patient care [12]. In recent years, the focus of research on symptom management in cancer patients has gradually shifted from evaluating individual symptoms to exploring the interaction patterns between symptoms. This shift in perspective has arisen because multiple symptoms often coexist and may be interconnected through complex mechanisms, thereby exacerbating the overall symptom burden on patients. The synergistic effects or interactions between multiple symptoms are referred to as “symptom clusters,” and the aims of the existing research have been to uncover the underlying mechanisms of these interrelationships [13, 14]. However, traditional symptom analysis methods, such as linear regression or correlation analysis, are often limited to univariate or isolated analyses of symptoms, making it difficult to comprehensively reveal the complex relationships and network structures between symptoms [15]. Research paradigms exploring the structural relationships among clinical symptoms are continuously evolving. These include the use of structural equation modeling (SEM) to investigate the latent structures and causal relationships between symptoms [16], the application of machine learning algorithms (such as random forests and support vector machines) and data mining techniques to identify complex relationships among symptoms [17], and the utilization of symptom network analysis to study the interrelationships between symptoms and identify core symptoms along with their association strengths with other symptoms.

Symptom network analysis is an emerging research method derived from network analysis, which is a method used to represent and understand the relationships between variables through graphical models. In a network graph, “nodes” represent different variables, whereas “edges” represent the relationships between variables (such as correlations or causal relationships). The advantage of network analysis is that it allows for simultaneous consideration of multiple relationships between variables and presents their overall structure rather than merely analyzing the impact of a single variable on others in isolation [18, 19]. In symptom network analysis, symptoms are treated as nodes in the network, and the associations between symptoms are treated as edges. The strength of the edges represents the degree of association between two symptoms, providing a clear representation of the complex relationships between multiple symptoms [20–22]. This method helps to identify core symptoms and their contribution to the overall symptom network. Through symptom network analysis, not only can the strength of associations between symptoms be quantified, but structural features of the symptom network, such as node centrality and network density, can also be identified, thereby providing scientific evidence for precision clinical interventions.

On the basis of the above background, the aim of this study was to construct and analyze the postoperative symptom network of NSCLC patients to reveal the complex patterns of associations among their postoperative symptoms and identify the core symptoms within the network. The results provide scientific evidence for precise symptom management and the development of personalized intervention strategies, thereby reducing the symptom burden on patients and improving their postoperative quality of life.

## Methods

### Study design and setting

This was a cross-sectional study investigating the core symptoms of postoperative thoracoscopic lung cancer patients. The reporting of this study conforms to the Strengthening the Reporting of Observational Studies in Epidemiology (STROBE) statement [23]. The participants were from the Department of Thoracic Surgery at a tertiary hospital in Chongqing, China. The study was approved by the Ethics Committee of the First Affiliated Hospital of Army Medical University (Approval No: (A) KY2024127), and all participants signed informed consent forms and voluntarily participated in the study. Although this is an observational study, the protocol was retrospectively registered (ChiCTR2500096720) after the completion of data analysis to align with open science principles.

### Study participants

In total, 367 postoperative NSCLC patients who were treated at the Department of Thoracic Surgery of the First Affiliated Hospital of Army Medical University between September and December 2024 were included in the study. The inclusion criteria were as follows: (1) Patients who underwent surgical treatment for lung cancer; (2) Age ≥ 18 years; (3) Fully conscious, able to communicate normally, and capable of reading; and (4) Patients diagnosed with NSCLC on the basis of pathological examination. The exclusion criteria were as follows: (1) Patients with recurrent lung cancer or distant organ metastasis; (2) Those receiving palliative care; (3) Patients with coexisting malignant tumors requiring treatment; or (4) Patients with severe disease in major organs.

### Sample size

There is currently no consensus on sample size calculation methods for network analysis. The scale used in this study includes 19 symptoms. According to the formula [N + N × (*N* − 1) / 2)] [24]. Constructing the network model requires the estimation of 19 threshold parameters and 171 pairwise association parameters [19 × (19 − 1) / 2], totaling 190 parameters. To ensure the reliability of the model, the sample size should be at least equal to the number of parameters. Considering a 20% non-response rate, the minimum required sample size is 228 cases. Ultimately, 367 lung cancer patients were included as study subjects.

### Data collection procedures and tools

The eligible research participants were required to sign a written informed consent form before data collection. The survey was administered to the participants by members of the research team. The survey included the following sections. Before the formal survey, training was provided to three investigators, and standardized survey terminology was established. This study used a paper-based questionnaire that was completed onsite. The informed consent form was attached to the front page of the questionnaire. After obtaining consent from the participant, the investigator provided a detailed explanation of the survey’s purpose, content, and research significance and then proceeded with the survey. Self-reported symptom scores were collected from patients 2 to 4 days after surgery. For each participant, the completed questionnaire was collected immediately after completion. Two data entry personnel entered all paper questionnaires into the database, with a third person verifying the data to ensure accuracy.

### Sociodemographic and clinical data

The sociodemographic information survey was independently designed and includes the following sections: (1) general demographic information: age, sex, marital status, employment status, education level, etc.; (2) disease-related information: duration of illness, pathology, TNM classification, etc.; and (3) surgical-related information: extent of surgical resection, duration of surgery, surgical site, etc.

### Chinese version of the MD Anderson symptom inventory

The MD Anderson Symptom Inventory (MDASI) was developed by the MD Anderson Cancer Center in the United States in 2000 to assess the severity of cancer symptoms or the impact of treatments. It consists of two sections: 13 symptom items and 6 symptom interference items [25]. In 2004, Wang et al. [26] translated and validated the MD Anderson Symptom Inventory for use with cancer patients in China, and the internal consistency reliability of the Chinese version ranged from 0.82 to 0.94. All the items were scored via a numeric rating scale, with scores ranging from 0 to 10. A score of 0 indicated no symptoms (i.e., the symptom did not occur), whereas scores from 1 to 10 indicated the occurrence of symptoms, with 10 representing the highest severity. Higher scores indicated more severe symptoms. In this study, the first section of the 13 core symptom items was used to assess the severity of symptoms in patients after lung cancer surgery.

## Revised lung cancer-specific symptom module

In 2011, the MD Anderson Cancer Center developed a lung cancer-specific module (MD Anderson Symptom Inventory-Lung Cancer module, MDASI-LC). The MDASI-LC includes 13 core symptoms from the original module and 3 lung cancer-specific symptoms (cough, constipation, and sore throat) [27]. However, a study by Zhang Lili et al. [28] reported that the symptoms included in this lung cancer module might not fully reflect the situation of lung cancer patients in China. Therefore, they made appropriate revisions to the MDASI-LC and validated its good psychometric properties in the Chinese lung cancer population. The revised scale includes 6 lung cancer-specific symptoms (cough, sputum production, hemoptysis, chest tightness, constipation, and weight loss), with a content validity of 0.773 and a construct validity of 0.922. Like in the core module, all the items are rated on a numeric scale from 0 to 10.

### Data analysis

#### Descriptive analysis

Statistical analysis of demographic and clinical characteristics was conducted via R software version 4.4.2. The percentage, standard deviation (SD), median (M), and interquartile range (IQR, P25, P75) were used to describe demographic and clinical characteristics, as well as symptom prevalence and severity. Multiple linear regression analysis was conducted to explore factors associated with overall symptom severity in postoperative non–non-small-cell lung cancer patients. A p-value of < 0.05 was considered to indicate statistical significance.

#### Contemporaneous symptom network analysis

Statistical analyses were performed with R version 4.4.2, with a significance level of α = 0.05. The “qgraph” package in R was used to construct a visualized network. A symptom network was built on the basis of the EBICglasso function and Spearman correlation analysis [29]. The network layout employed the spring layout, where symptoms were represented as nodes and the connections between nodes represented edges. The thicker the edge was, the stronger the correlation between the two symptoms.

The centrality indices included strength centrality, closeness centrality, and betweenness centrality [30]. Strength centrality represents the weighted sum of all edges connected to a node, measuring the importance of a node in the network. Closeness centrality is the reciprocal of the sum of the shortest path distances from the node to all other nodes in the network. Betweenness centrality is the frequency at which a node lies on the shortest path between any two other nodes, helping to identify core symptoms [31].

The “bootnet” package in R was used to estimate 95% confidence intervals via the bootstrap algorithm to evaluate the accuracy of the edge weights and centrality indices. The correlation stability coefficient was calculated, where a value greater than 0.25 indicates acceptable network model stability and a value greater than 0.50 indicates good network model stability. A p-value < 0.05 was considered statistically significant [29].

## Results

### Characteristics of the participants

This study distributed 404 questionnaires, and after data authenticity verification, 367 valid questionnaires were returned, resulting in an effective response rate of 90.8%. The average age of the 367 patients was 58.92 ± 9.74 years. Other general and surgical-related information can be found in Table 1.

**Table 1:** Characteristics of the participants (*n* = 367)

Characteristics	*n* (%), M ± SD
Age	58.92 ± 9.74
Sex	
Male	162 (44.1)
Female	205 (55.9)
Occupation	
Employed	99 (27.0)
Formerly employed	5 (1.4)
Retired	115 (31.3)
Unemployed	148 (40.3)
Payment Method	
Cooperative medical insurance	182 (49.6)
Employee health insurance	166 (45.2)
Commercial insurance	13 (3.6)
Out-of-pocket	6 (1.6)
Marital Status	
Married	343 (93.4)
Widowed	12 (3.3)
Divorced	11 (3.0)
Single	1 (0.3)
Education Level	
Primary school or below	134 (36.5)
Junior high school	107 (29.2)
Senior high school/technical school	61 (16.6)
College or above	65 (17.7)
Monthly Household Income	
< 3,000 RMB	197 (53.7)
3,000–5,000 RMB	108 (29.4)
5,000 RMB	62 (16.9)
Residence	
Urban	230 (62.7)
Rural	137 (37.3)
Smoking History	
Yes	126 (34.3)
No	241 (65.7)
BMI (kg/m^2^)	
< 18.5	8 (2.2)
18.5 ~ 23.9	175 (47.7)
24.0 ~ 27.9	115 (31.3)
≥ 28.0	69 (18.8)
Comorbidities	
Yes	115 (31.3)
No	252 (68.7)
Pathological Type	
Adenocarcinoma	336 (91.6)
Squamous carcinoma	31 (8.4)
TNM Classification	
I	313 (85.3)
II	30 (8.2)
III	21 (5.7)
IV	3 (0.8)
Disease Duration	
< 3 months	181 (49.3)
3–6 months	55 (15.0)
> 6 months	131 (35.7)
Surgical Method	
Wedge resection	155 (42.2)
Radical lung cancer surgery	89 (24.3)
Lung segmentectomy	88 (24.0)
Lobectomy	35 (9.5)
Surgical Duration	
< 2 h	142 (38.7)
2–4 h	217 (59.1)
>4 h	8 (2.2)
Surgical Site	
Left	150 (40.9)
Right	217 (59.1)

Note: M: mean; SD: standard deviation; BMI: body mass index; RMB: renminbi. Comorbidities refer to whether the patient has chronic conditions such as diabetes, hypertension, chronic obstructive pulmonary disease, or other similar diseases

### Symptom prevalence and severity

The symptom prevalence and severity in patients with non-small cell lung cancer are shown in Table 2. The most prevalent symptom was pain (*n* = 367, 100%), followed by fatigue (*n* = 362, 98.64%), shortness of breath (*n* = 361, 98.37%), and cough (*n* = 360, 98.09%). The most severe symptom was pain (4.05 ± 1.53), followed by fatigue (3.77 ± 1.99), shortness of breath (2.63 ± 1.53), and cough (2.29 ± 1.17). Symptoms with a postoperative incidence rate of ≤ 20% included numbness (7.36%) and vomiting (17.44%).

**Table 2:** Symptom prevalence and severity in the participants (*n* = 367)

Classification	Items	Symptoms	Prevalence [n (%)]	Symptom severity		
				Mean (SD)	M (P_25,_ P_75_)	
MDASI	S1	Pain	367 (100.00)	4.05 (1.53)		4 (3,5)
	S2	Fatigue	362 (98.64)	3.77 (1.99)		3 (2.5,5)
	S3	Nausea	75 (20.44)	0.23 (0.52)		0 (0,0)
	S4	Disturbed sleep	355 (96.73)	1.96 (1.13)		2 (1,2)
	S5	Distress	181 (49.32)	0.55 (0.67)		0 (0,1)
	S6	Shortness of breath	361 (98.37)	2.63 (1.53)		2 (2,3)
	S7	Difficulty remembering	226 (61.58)	0.93 (0.94)		1 (0,1)
	S8	Appetite loss	335 (91.28)	1.56 (1.03)		1 (1,2)
	S9	Drowsiness	286 (77.93)	0.96 (0.71)		1 (1,1)
	S10	Dry mouth	274 (74.66)	1.08 (0.85)		1 (0,2)
	S11	Sadness	168 (45.78)	0.49 (0.57)		0 (0,1)
	S12	Vomiting	64 (17.44)	0.21 (0.49)		0 (0,0)
	S13	Numbness	27 (7.36)	0.95 (0.37)		0 (0,0)
MDAS-LC	S14	Cough	360 (98.09)	2.29 (1.17)		2 (1,3)
	S15	Expectoration	334 (91.01)	1.74 (1.10)		2 (1,2)
	S16	Hemoptysis	238 (64.85)	0.74 (0.62)		1 (0,1)
	S17	Chest tightness	253 (68.94)	1.05 (0.97)		1 (0,2)
	S18	Constipation	303 (82.56)	1.38 (1.12)		1 (1,2)
	S19	Weight loss	95 (25.89)	0.27 (0.46)		0 (0,1)

Note: M: mean; SD: standard deviation; P_25_, P_75_ represents Interquartile range

### Factors associated with overall symptom severity

Table 3 shows the results of the multivariable linear regression analysis for overall symptom severity. Female sex (*P* = 0.004), smoking history (*P* = 0.050), and lobectomy (*P* = 0.002) were significantly associated with the symptom severity score.

**Table 3:** Linear regression model of overall symptom severity (*n* = 367)

Characteristics	β		*P*
Age	0.005	0.080	
Sex (compared to male)	0.230	**0.004**	
Smoking History (compared to No)	0.164	**0.050**	
Chronic Disease History (compared to No)	0.054	0.375	
BMI (compared to overweight)			
Underweight (< 18.5)	-0.001	0.998	
Normal (18.5–23.9)	0.065	0.305	
Obesity (≥ 28.0)	-0.146	0.085	
Pathological Type (compared to squamous carcinoma)	-0.036	0.733	
TNM Classification (compared to stage II)			
I	0.117	0.271	
II	-0.005	0.971	
III	0.028	0.930	
Disease Duration (compared to < 3 months)			
3–6 months	-0.014	0.862	
> 6 months	-0.066	0.279	
Surgical Method (compared to Radical lung cancer surgery)			
Wedge resection	0.011	0.886	
Lobectomy	-0.108	0.199	
Lung Segmentectomy	0.329	**0.002**	
Surgical Duration (compared to < 2 h)			
2–4 h	-0.008	0.898	
> 4 h	0.206	0.290	

Note: R² = 0.114; adjusted R² = 0.062; F = 2.214; *P* = 0.002; BMI: body mass index; bold values indicate statistical significance at *P* < 0.05

### Symptom network and centrality indicators

**Fig. 1:**
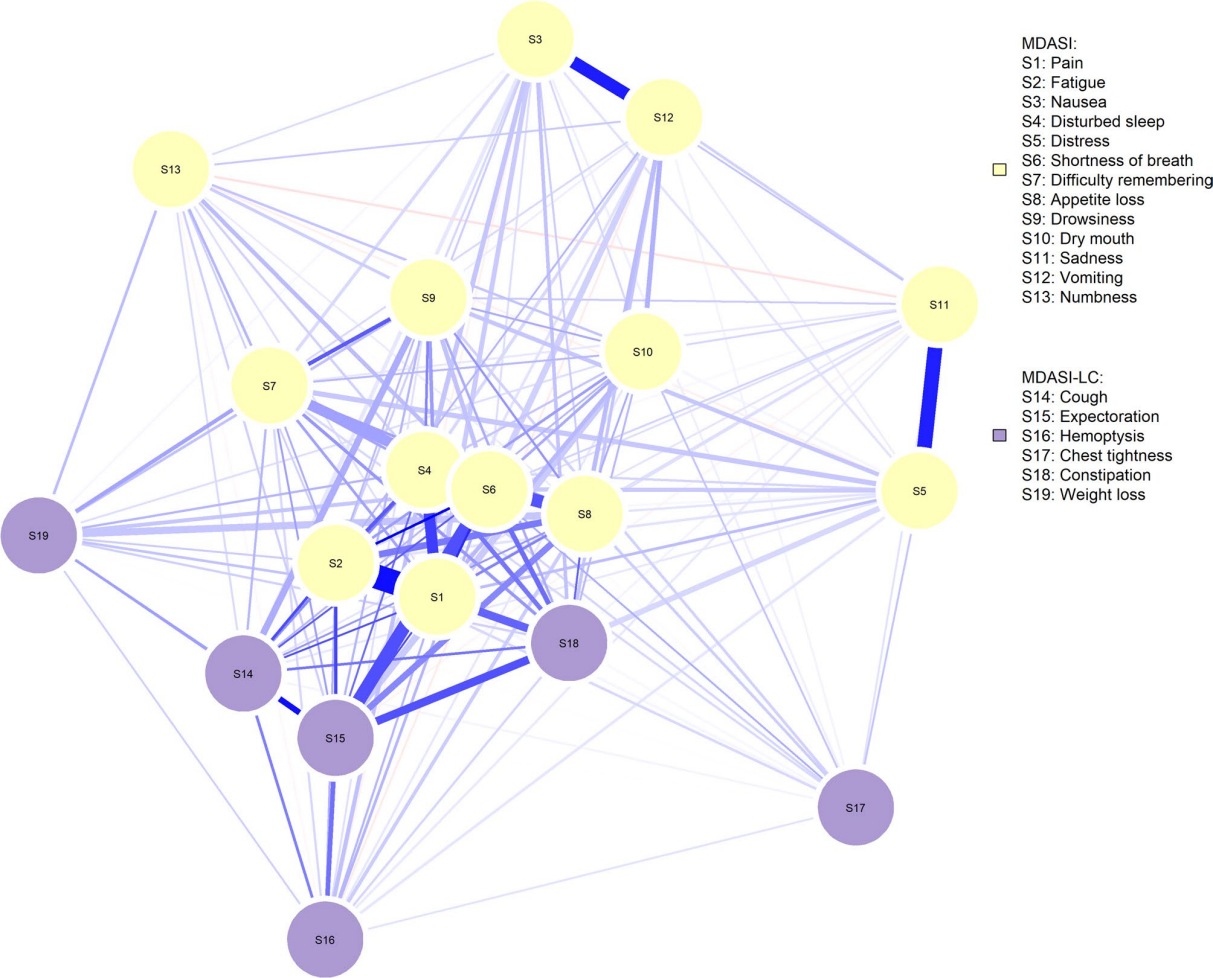
Postoperative Symptom Network in Patients with NSCLC. **Note**: The symptom network layout was generated using the Fruchterman–Reingold algorithm, which clusters more strongly connected nodes together to estimate the optimal layout. In the symptom network graph, circular nodes represent symptoms, and edges between nodes represent correlations. The thickness of the edges indicates the strength of the correlation between nodes, with blue edges representing positive correlations and red edges representing negative correlations. MDASI: Chinese version of the MD Anderson Symptom Inventory; MDASI-LC: Revised lung cancer-specific symptom module

In the postoperative symptom network of patients with NSCLC, strong associations were observed between pain and shortness of breath, pain and fatigue, pain and sleep disturbances, nausea and vomiting, and distress and sadness. Pain is connected to multiple nodes, indicating a strong relationship between pain and other symptoms (Fig. 1).

**Fig. 2:**
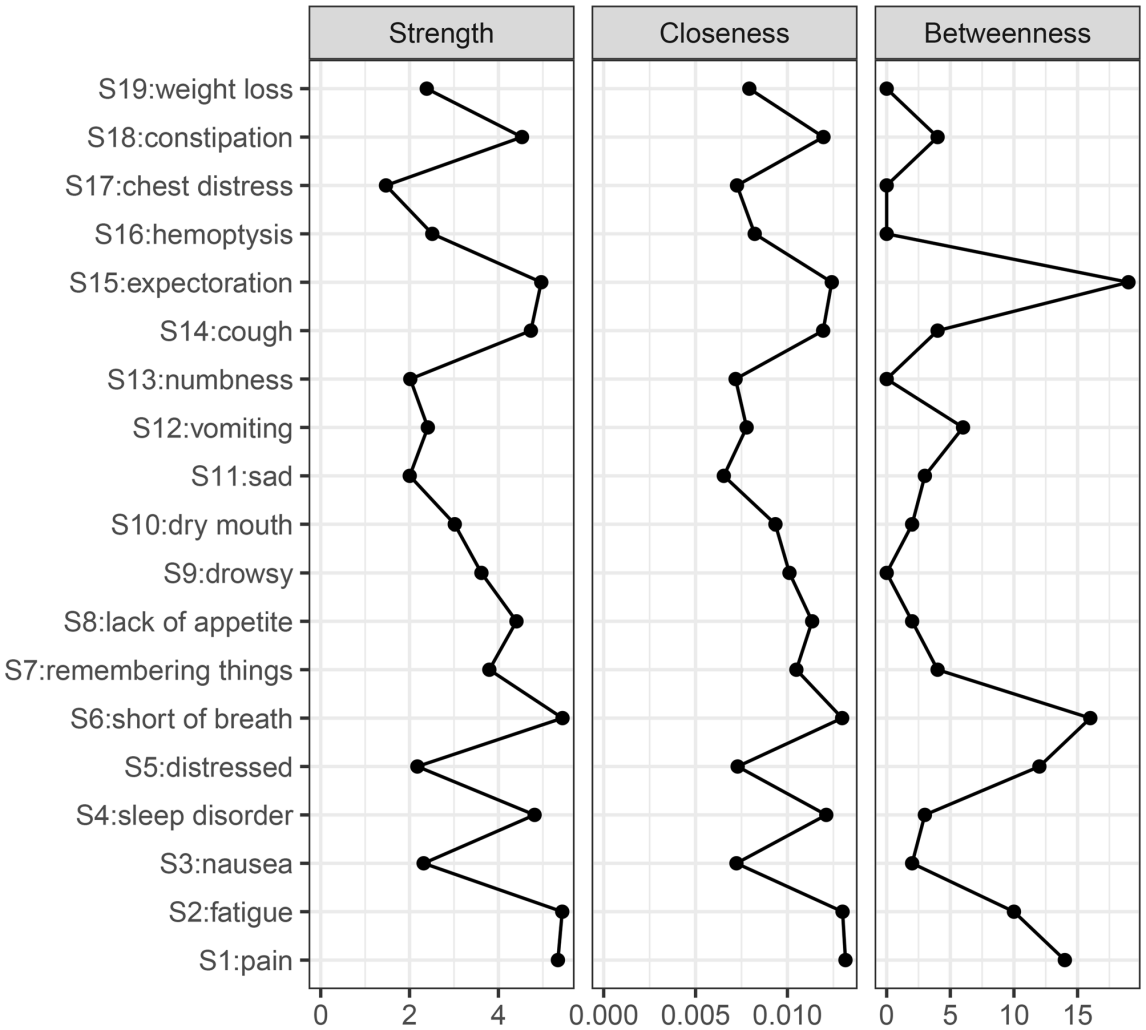
Node Centrality Indicators in the Postoperative Symptom Network of Patients with NSCLC. **Note**: The Y-axis represents different symptoms, and the X-axis represents the scores of these symptoms on specific centrality indicators

The results of the centrality indicators revealed that the top three symptoms in terms of strength centrality were shortness of breath (r_s_ = 5.44), fatigue (r_s_ = 5.43), and pain (r_s_ = 5.34). The top three symptoms in terms of closeness centrality were pain (r_c_ = 0.0132), shortness of breath (r_c_ = 0.0130), and fatigue (r_c_ = 0.0130). The top three symptoms in terms of betweenness centrality were cough (r_b_ = 38), shortness of breath (r_b_ = 32), and pain (r_b_ = 28) **(**Fig. 2**).**

### Edge weight accuracy and node stability

**Fig. 3:**
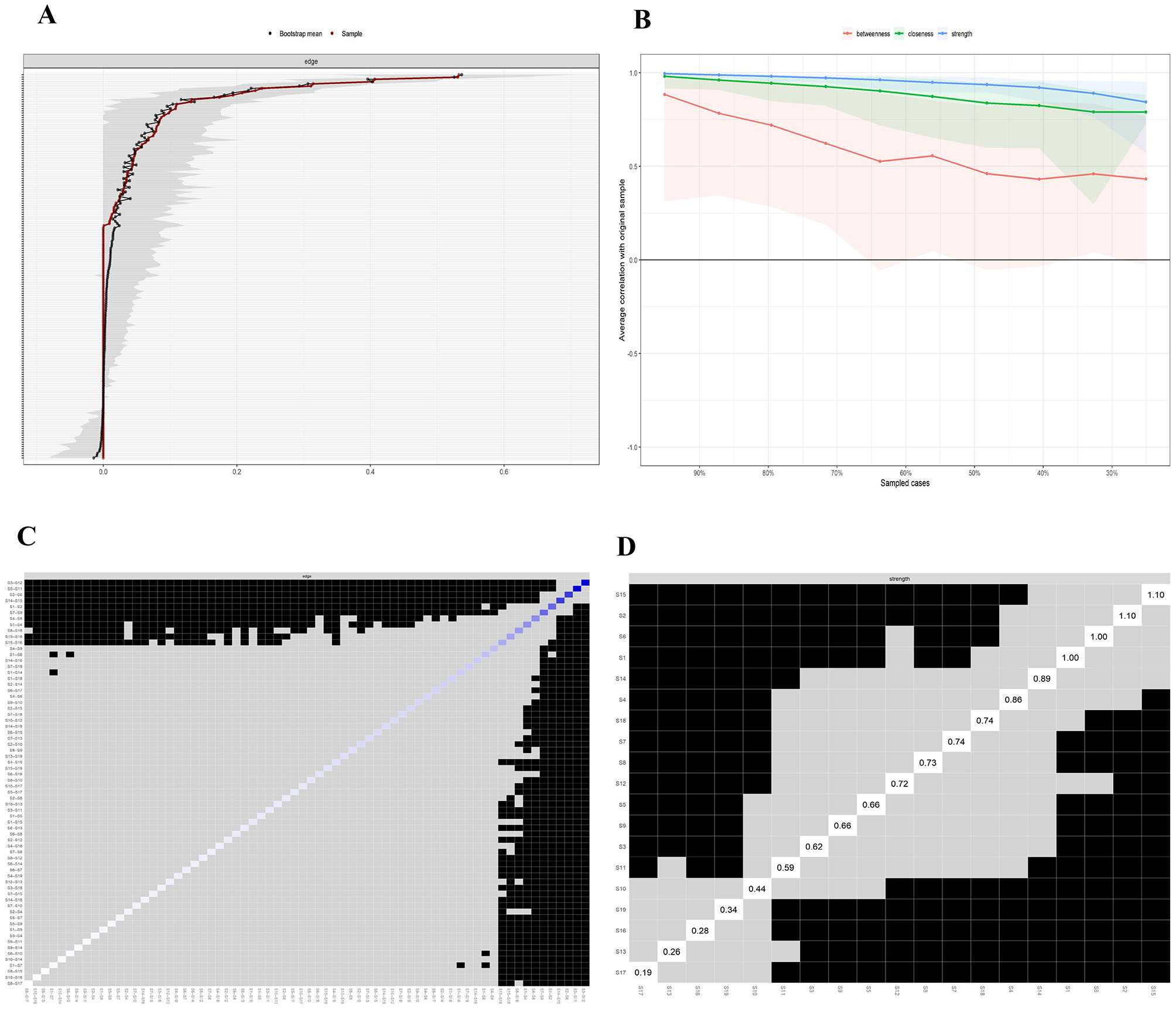
Accuracy, Stability, and Bootstrap Difference Test of Symptom Network Edge Weights. **A**: Edge weight accuracy test; The grey area represents the bootstrap 95% confidence interval, which was narrow and indicated high accuracy. **B**: Node stability test; Case-dropping bootstrap procedure for stability of centrality indices in terms of strength, betweenness and closeness. **C**: Edge weight difference test; **D**: Node difference test; Gray boxes indicate nodes or edges that do not differ significantly and black boxes represent nodes or edges that differ significantly

The weighted values of the edges in this sample closely align with the 95% confidence intervals (CIs) of the edge weights obtained via the bootstrap method, with relatively narrow confidence intervals. This indicates that the edge weights have sufficient accuracy, meaning that the effect estimates of these edges are stable and reliable **(**Fig. 3A**)**. The stability test results revealed that the intensity centrality and expected impact stability coefficients calculated via the case‒dropping bootstrap method were both 0.673, greater than 0.50, indicating good network stability. Even with a reduced sample size, the core symptoms remained stable **(**Fig. 3B**).**

### Test of edge weights and node differences

The bootstrap method was used to test the differences in edge weights. The black squares indicate that the two edges are significantly different, whereas the gray squares indicate that there is no significant difference between the edges. The edge weights of nausea and vomiting (S3–S12), distress and sadness (S5–S11), and fatigue and shortness of breath (S2–S6) were significantly different (*P* < 0.05) from those of most other edge weights, suggesting that the highly correlated symptom edge weights were significantly higher than the other edge weights were **(**Fig. 3C**).** The bootstrap method was also used to test the differences in expected node influence. The expected influences of the nodes constipation (S15), pain (S2), and shortness of breath (S6) were significantly different (*P* < 0.05) from the expected influences of other nodes **(**Fig. 3D**)**.

## Discussion

### Shortness of breath is the core symptom in postoperative NSCLC patients

In this study, we identified shortness of breath as a core symptom in NSCLC patients after surgery. Shortness of breath refers to the sensation of difficulty breathing, either at rest or during activity, and typically manifests as rapid, labored, or obstructed breathing, often accompanied by chest tightness and the inability to breath fully [32, 33]. The results of several studies have indicated [34–36] that shortness of breath is very common in lung cancer patients after surgery, especially among those undergoing lobectomy, where the incidence is even more pronounced. A study by Alessandro Brunelli and colleagues on 152 long-term survivors of early-stage lung cancer after minimally invasive segmental resection or lobectomy revealed that 82% of lobectomy survivors reported worsening shortness of breath, compared to 57% who underwent segmental resection [35]. Similarly, our multiple linear regression analysis revealed that patients who underwent lobectomy had higher overall symptom severity scores than those who underwent segmental or wedge resections did. Furthermore, we found that female patients experienced more pronounced shortness of breath than male patients did, which is consistent with the findings of Cecilia Pompili and colleagues [37]. Extensive lobectomy or pneumonectomy can lead to a decline in lung function and reduced lung capacity, thus affecting the efficiency of gas exchange [38, 39]. Postoperative pulmonary complications, such as pneumonia, atelectasis, and pleural effusion, can also directly impact gas exchange, leading to shortness of breath. In line with this, a dynamic network analysis indicated that fatigue is the most central symptom on postsurgical days 1–2 and that shortness of breath becomes the most central symptom on postsurgical days 5–6 [40]. In our study, we surveyed patients on postsurgical days 3–5, which somewhat aligns with these findings. Shortness of breath is not observed only during hospitalization, as research has confirmed that it persists after discharge. A longitudinal cohort study monitoring symptoms in lung cancer patients post-surgery revealed that cough and shortness of breath ranked among the top two symptoms in terms of severity after discharge [41]. A study on APP-based symptom management revealed that the most common symptom alerts for lung cancer patients were pain (40.6%) and shortness of breath (28.1%) at two weeks and one month post-surgery [42]. This suggests that we need to focus on managing shortness of breath not only during hospitalization but also after discharge, as shortness of breath is one of the main contributors to the symptom burden affecting the quality of life of patients [43]. Postoperative shortness of breath not only impacts quality of life but also may be closely related to prognosis. A prospective cohort study revealed that shortness of breath on the first day after surgery can serve as an early warning for postoperative pulmonary complications. A self-reported shortness of breath score greater than or equal to 6 may indicate the occurrence of pulmonary complications [44]. Another study indicated that shortness of breath on the day of discharge can serve as a warning for complications within three months post discharge. Patients reporting a shortness of breath score of 5 or higher on the day of discharge are more likely to experience complications within three months [45]. Therefore, our findings are consistent with those of several studies, and by using symptom network analysis, we have strengthened the existing evidence, supporting the clinical observation that shortness of breath is a central symptom in the symptom network of lung cancer patients post-surgery.

### Pain as a key symptom in the symptom network

Our study results indicate that the incidence and severity of pain are very high, highlighting that pain is also a major symptom that requires focused attention after surgery in NSCLC patients. In a multicenter cross-sectional study involving 533 lung cancer patients, 45.4% of patients were reported to have experienced pain during their treatment, and 24.2% of patients reported experiencing pain in the last 24 h [46]. Furthermore, in the study by Wang S et al.‘s [47] the incidence of moderate-to-severe pain within 24 h post-surgery was reported to be 28.3%, and age was found to influence postoperative pain occurrence. In particular, patients younger than 58.5 years who underwent curative surgery for lung cancer were more likely to experience moderate-to-severe pain within 24 h post-surgery. Furthermore, a significant negative correlation was found between postoperative pain scores at 24 h and good or excellent postoperative recovery [48]. Our study also revealed varying degrees of pain in lung cancer patients during hospitalization. Patients often present with acute pain, with some developing persistent pain. The overall prevalence of persistent pain after thoracic surgery is 38.1% [49], and persistent postoperative pain is closely associated with the occurrence of postoperative complications [50]. Persistent pain can further evolve into chronic pain, and in their study, Clephas PRD et al. [51] identified acute pain, increased postoperative pain intensity, and female sex as risk factors for the development of chronic postoperative pain. Common sites of pain one month after lung cancer surgery include the chest (43%), neck/shoulders (36%), and back (32%) [52].

In the symptom network diagram, a strong association was observed between postoperative pain, fatigue, shortness of breath, and sleep disturbances. Several studies have included pain, fatigue, and sleep disturbances in the same symptom cluster for management [53, 54], reasoning that their mechanisms share similarities, allowing for centralized management. Pain can decrease the quality of sleep, and poor sleep quality further exacerbates fatigue, illustrating the interrelationship between pain, fatigue, and sleep disturbances. The trajectory of symptoms and fatigue in patients is influenced by pain, functional status decline, total symptom scores, and depression [55]. Our findings also revealed a strong association between pain and shortness of breath. Severe pain after chest surgery limits deep breathing and effective coughing, making it more difficult to expel lung secretions and potentially leading to further lung function impairment, which in turn exacerbates shortness of breath. Relaxation therapy has been shown to effectively reduce both breathing difficulty and pain severity while improving sleep quality [56]. Our findings, presented through the symptom network diagram, provide a more intuitive view of how pain is associated with other symptoms and the degree of these associations.

### Strengths and limitations

This study revealed the complex interrelationships between symptoms in NSCLC patients through network analysis, providing a comprehensive reflection of symptom interaction patterns in the real world. The identification of shortness of breath as a core symptom offers theoretical support for precise symptom management. Furthermore, the unique perspective of symptom network analysis opens new avenues for managing multi-symptom diseases and promotes the precise design of symptom management strategies.

However, there are certain limitations in this study. First, the use of a single-center sample may affect the external validity of the results, limiting their generalizability across different regions. Second, the symptom checklist used in the study covered only a limited number of common symptoms, potentially overlooking some unique symptoms experienced by individual patients, thus affecting the completeness of the study. Finally, owing to the cross-sectional design and convenience sampling method, the study was unable to explore causal relationships between symptoms in depth, which limits the broader applicability of the results and a deeper understanding of the mechanisms of symptom associations. Therefore, future research should adopt multicenter, large-sample longitudinal designs to explore causal relationships between symptoms and their changes over time. Conduct dynamic network analyses at multiple postoperative time points to capture the evolution of symptoms and their interrelationships over time. When conducting multi-center longitudinal studies, it is necessary to ensure that the samples are representative, determine the time points reasonably, and standardize the symptom checklist.

### Implications for nursing practice and research

The results of this study indicate that shortness of breath is a core symptom in NSCLC patients after surgery and that it may signal impaired lung function or the risk of postoperative complications. Clinical nursing staff should closely monitor patients’ respiratory conditions, promptly identify early signs of shortness of breath, and implement targeted interventions to reduce postoperative complications and improve patient prognosis and quality of life. Additionally, shortness of breath not only impacts patients’ physiological functions but also severely affects their daily activities and psychological well-being. Nursing staff should address both physiological and psychological needs, develop personalized care plans, and implement supportive treatments such as oxygen therapy, psychological therapy, or pulmonary rehabilitation to alleviate symptoms and promote recovery.

Moreover, the results of the study revealed a strong correlation between pain and symptoms such as shortness of breath and fatigue, highlighting the complexity of symptom management. Nursing staff should consider the interrelationships among symptoms comprehensively and adopt a holistic intervention strategy. Through multidisciplinary collaboration, continuous monitoring, and the dynamic adjustment of care measures, the interactions between symptoms can be alleviated, optimizing the overall recovery process. This integrated nursing intervention will significantly increase the quality of postoperative recovery and provide more precise and comprehensive nursing care for patients.

## Conclusions

Our study established a symptom network for NSCLC patients during postoperative hospitalization and explored the interrelationships between symptoms by means of network analysis. By calculating centrality indices and network density, we revealed strong connections between symptoms, providing theoretical support for precise symptom management strategies. On the basis of postoperative symptom data from 367 NSCLC patients, we identified shortness of breath as the core symptom in the network, highlighting its clinical significance as a priority intervention target. These findings provide scientific evidence for personalized treatment and symptom management, helping reduce symptom burden and improve quality of life. Future research should focus on dynamic symptom networks, with longitudinal data and trajectory analysis of centrality indices helping reveal causal relationships between symptoms, further optimizing clinical interventions and promoting individualized treatment strategies.

## Data Availability

The data used in our study are available from the corresponding author upon reasonable request.

## Abbreviations

NSCLC: Non-Small Cell Lung Cancer
BMI: Body Mass Index
MDSCI: MD Anderson Symptom Inventory
MDSCI-LC: MD Anderson Symptom Inventory-Lung Cancer module
